# Detection of Clinically Significant BRCA Large Genomic Rearrangements in FFPE Ovarian Cancer Samples: A Comparative NGS Study

**DOI:** 10.3390/genes16091052

**Published:** 2025-09-08

**Authors:** Alessia Perrucci, Maria De Bonis, Giulia Maneri, Claudio Ricciardi Tenore, Paola Concolino, Matteo Corsi, Alessandra Conca, Jessica Evangelista, Alessia Piermattei, Camilla Nero, Luciano Giacò, Elisa De Paolis, Anna Fagotti, Angelo Minucci

**Affiliations:** 1 Departmental Unit of Molecular and Genomic Diagnostics, Fondazione Policlinico Gemelli IRCCS, 00168 Rome, Italy; alessia.perrucci@guest.policlinicogemelli.it (A.P.); maria.debonis@policlinicogemelli.it (M.D.B.); giulia.maneri@guest.policlinicogemelli.it (G.M.); claudio.ricciarditenore@guest.policlinicogemelli.it (C.R.T.); paola.concolino@policlinicogemelli.it (P.C.); matteo.corsi@guest.policlinicogemelli.it (M.C.); alessandra.conca@guest.policlinicogemelli.it (A.C.); jessica.evangelista@policlinicogemelli.it (J.E.); elisa.depaolis@policlinicogemelli.it (E.D.P.); 2 Genomics Core Facility, G-STeP, Fondazione Policlinico Universitario Agostino Gemelli IRCCS, 00168 Rome, Italy; 3 Pathology Unit, Department of Woman and Child’s Health and Public Health Sciences, Fondazione Policlinico Universitario Agostino Gemelli IRCCS, 00168 Rome, Italy; alessia.piermattei@policlinicogemelli.it; 4 Unit of Oncological Gynecology, Department of Women, Children and Public Health Sciences, Fondazione Policlinico Universitario Agostino Gemelli IRCCS, 00168 Rome, Italy; camilla.nero@policlinicogemelli.it (C.N.); anna.fagotti@policlinicogemelli.it (A.F.); 5 Department of Women, Children and Public Health Sciences Università Cattolica del Sacro Cuore, 00168 Rome, Italy; 6 Bioinformatics Research Core Facility, Gemelli Science and Technology Park (G-STeP), Fondazione Policlinico Universitario Agostino Gemelli IRCCS, 00168 Rome, Italy; luciano.giaco@policlinicogemelli.it

**Keywords:** *BRCA* genes, NGS, large genomic rearrangements, ovarian cancer, copy number variations, FPG500, *Diatech Myriapod^®^ NGS BRCA1/2* panel kit, SOPHiA DDM™ Homologous Recombination Solution

## Abstract

Background: Copy number variations (CNVs), also referred to as large genomic rearrangements (LGRs), represent a crucial component of *BRCA1/2 (BRCA)* testing. Next-generation sequencing (NGS) has become an established approach for detecting LGRs by combining sequencing data with dedicated bioinformatics pipelines. However, CNV detection in formalin-fixed paraffin-embedded (FFPE) samples remains technically challenging, and there is the need to implement a robust and optimized analysis strategy for routine clinical practice. Methods: This study evaluated 40 FFPE ovarian cancer (OC) samples from patients undergoing *BRCA* testing. The performance of the amplicon-based NGS *Diatech Myriapod^®^ NGS BRCA1/2* panel (Diatech Pharmacogenetics, Jesi, Italy) was assessed for its ability to detect *BRCA* CNVs and results were compared to two hybrid capture-based reference assays. Results: Among the 40 analyzed samples (17 CNV-positive and 23 CNV-negative for *BRCA* genes), the Diatech pipeline showed a good concordance with the reference method—all CNVs were correctly identified in 16 cases with good enough sequencing quality. Only one result was inconclusive due to low sequencing quality. Conclusions: These findings support the clinical utility of NGS-based CNV analysis in FFPE samples when combined with appropriate bioinformatics tools. Integrating visual inspection of CNV plots with automated CNV calling improves the reliability of CNV detection and enhances the interpretation of results from tumor tissue. Accurate CNV detection directly from tumor tissue may reduce the need for additional germline testing, thus shortening turnaround times. Nevertheless, blood-based testing remains mandatory to determine whether detected *BRCA* CNVs are of hereditary or somatic origin, particularly in cases with a strong clinical suspicion of inherited predisposition due to young age and a personal and/or family history of OC.

## 1. Introduction

Hereditary breast and/or ovarian cancer syndrome has traditionally been the primary criterion for genetic counseling, followed by germline *BRCA1/2 (BRCA)* testing [1]. However, over the past decade, numerous studies have demonstrated that ovarian cancer (OC) patients harboring germline or somatic pathogenic *BRCA* variants (PVs) show sensitivity to poly (ADP-ribose) polymerase inhibitors (PARPi) and platinum-based chemotherapy [2,3]. In addition, functional defects in homologous recombination repair genes, collectively referred to as homologous recombination deficiency (HRD), have been clinically validated as predictive biomarkers for PARPi treatment in OC [4]. As a result, *BRCA* and/or HRD testing on formalin-fixed paraffin-embedded (FFPE) tumor samples, which allow simultaneous detection of both somatic and germline PVs, has become increasingly important in the molecular management of OC patients [5,6,7].

Copy number variations (CNVs), also referred to as large genomic rearrangements (LGRs), such as deletions or duplications larger than 1000 base pairs, have been identified in *BRCA* genes. Their prevalence varies widely among populations, ranging from less than 1% to more than 24% [8]. Consequently, LGRs account for a substantial proportion of *BRCA* PVs and are now an integral component of *BRCA* and HRD testing [6].

Next-generation sequencing (NGS) is now a well-established method for comprehensive *BRCA* screening from blood, enabling the simultaneous detection of single nucleotide variants (SNVs), insertions/deletions (*indels*), and CNVs [9,10,11]. However, CNV detection in tumor tissue presents specific challenges, including tumor heterogeneity, low tumor cellularity, the absence of a matched normal baseline, poor DNA quality, and the presence of PCR contaminants or artifacts. These factors can lead to uneven sequencing coverage across genomic regions, impairing the accurate identification of CNVs. As a result, NGS-based CNV detection may generate false positives or, more critically, false negatives, particularly when using workflows that lack validated and dedicated bioinformatics pipelines [10,11].

Among various NGS protocols, hybrid capture-based approaches have demonstrated greater reliability for CNV detection compared to amplicon-based PCR protocols. Nonetheless, several *BRCA* CNV assays are currently available, and not all of these are fully validated for clinical use or supported by robust bioinformatics pipelines [12].

The aim of this study was to evaluate the ability of different NGS bioinformatics pipelines to accurately identify and call *BRCA* CNVs from FFPE tumor samples. To this end, 40 OC samples were selected, including 17 samples harboring clinically significant LGRs. CNV calls from two hybrid capture-based NGS protocols were compared with those obtained using the amplicon PCR-based *Diatech Myriapod^®^ NGS BRCA1/2 panel* kit.

Finally, a strategy was proposed to improve the interpretation of NGS data for reliable identification of CNVs in FFPE samples in a clinical setting.

## 2. Materials and Methods

### 2.1. Study Design

This study was conducted at the Genomics Core Facility, G-STeP, Fondazione Policlinico Universitario Agostino Gemelli IRCCS. A total of 40 FFPE tumor samples from patients with advanced or relapsed platinum-sensitive OC were selected. All samples were previously screened for *BRCA* status in our center using specific approaches: 13 of these were tested using the *TruSight Oncology 500 High Throughput* (TSO500HT) (Illumina Inc., San Diego, CA, USA), according to the FPG500 program [13], 9 using the *SOPHiA DDM™ Homologous Recombination Solution* (SOPHiA DDM™ HRD) (SOPHiA GENETICS, Lausanne, Switzerland), and 18 underwent both procedures. Sample characteristics and molecular results are summarized in Table 1. As reported, 17 samples were *BRCA* CNV-positive and 23 were negative. In addition, 8 CNVs detected on tissue samples showed a germline origin.

To evaluate the performance of *BRCA* CNV calling using the *Diatech Myriapod^®^ NGS BRCA1/2* panel kit (Diatech Pharmacogenetics, Jesi, Ancona, Italy), the selected FFPE samples were retested with this assay. All participants provided informed consent to participate in the study (Study ID: FPG500; Ethics Committee Approval No. 3837), which was conducted in accordance with the Declaration of Helsinki.

### 2.2. DNA Extraction

For DNA extraction, FFPE tissue samples containing >20% tumor cells and <10% necrosis, as determined by the local pathologist, were selected. DNA was extracted using the Qiagen AllPrep DNA/RNA FFPE Kit on the EZ2 Connect workstation (Qiagen, Hilden, Germany), following the manufacturer’s protocol. DNA quantity and quality were assessed using the Qubit 3.0 Fluorometer (Thermo Fisher Scientific, Waltham, MA, USA).

### 2.3. NGS Methods Used for CNV Calling

The *TSO500HT* assay is a panel-based NGS assay that uses a hybridization–capture-based target enrichment strategy and assesses 523 cancer-related genes. In a single assay, it enables the detection of SNVs, *indels*, and CNVs of 523 genes, measures genomic signatures (including MSI and TMB), and supports RNA-based calling of fusions of 55 genes and alternative splicing events in FFPE tumor tissue samples [13].

CNV calling for the *TSO500HT* assay was conducted using the Dragen TSO500HT v2 software ((Illumina Inc., San Diego, CA, USA). CNV analysis includes 500 CNV associated genes and can call amplifications with a limit of detection at 2.2× fold change and deletions at 0.5× fold change. In addition, a CNV step in the Dragen TSO500HT analysis workflow enables exon-level CNV detection for *BRCA* genes. The tertiary analysis was performed using PierianDx CGW.

*SOPHiA DDM™ HRD* is a hybridization-based approach able to combine in a single assay the detection by targeted sequencing of SNVs and *indels* in 28 homologous recombination repair (HRR) genes, including *BRCA* genes, and the assessment of the Genomic Integrity Index (GII) for HRD status. CNV calling was performed using the *SOPHiA DDM™* platform on the coding regions of 28 genes, including the *BRCA* genes. For both NGS solutions, CNV calling is performed using a proprietary algorithm.

### 2.4. BRCA Testing with Myriapod^®^ NGS BRCA1/2 Panel Kit and Primary Sequencing Strategy

The *Myriapod^®^ NGS BRCA1/2* panel kit software (Diatech Pharmacogenetics, Jesi, Ancona, Italy) is an in vitro diagnostic assay that enables the detection of SNVs, *indels*, and splice variants in the *BRCA* genes.

CNV analysis was performed using the *Myriapod NGS* data analysis software version (v) 5.0.11, which uses a proprietary algorithm for CNV calling. CNV analysis is based on the evaluation of coverage variations of contiguous amplicons for each exon, after intra-run normalization of the single sample’s coverage. Normalized amplicon coverages are then compared to a global threshold to assess the presence of a potential CNV.

With the aim of evaluating the performance of the CNV detection algorithm of the *Myriapod^®^ NGS BRCA1/2* panel kit considering “stressed” testing conditions, a “primary CNV calling strategy” was defined. In this strategy, four NGS runs, each including 10 FFPE samples, were performed on the MiSeq System, using the MiSeq Micro Flow Cell (Illumina Inc., San Diego, CA, USA). Specifically, two runs included 5 *BRCA* CNV-positive and 5 *BRCA* CNV-negative cases, while the other two runs consisted of 4 *BRCA* CNV-positive and 6 *BRCA* CNV-negative samples, respectively.

### 2.5. Re-Evaluation of CNV Calling Using Diatech Software by Simulating a Diagnostic Setting

To further evaluate the CNV calling performance of the *Myriapod^®^ NGS BRCA 1-2* kit in association with the *Myriapod^®^ NGS* data analysis software (v 5.0.11), all 40 samples were re-analyzed to simulate a routine clinical setting. Specifically, the analysis was designed to reflect a scenario in which CNVs occur with an estimated prevalence of less than 10% in the general population, corresponding to the likelihood of detecting at most one positive case per sequencing run of 10 samples.

### 2.6. Read Coverage and Comparative Analyses

Sequencing performance was evaluated across the 4 NGS runs, with the aim of optimizing CNV calling. Key quality metrics assessed included mean coverage, percentage of uniformity, and on-target reads. Results were analyzed separately for each sequencing run and summarized as the mean ± standard deviation (SD) and confidence intervals, across all samples within each run.

In parallel with the assessment of sequencing quality, statistical analyses were conducted to evaluate CNV calling performance using the *Myriapod^®^ NGS BRCA1/2* panel kit in association with the *Myriapod^®^ NGS* data analysis software (v 5.0.11). The overall accuracy, sensitivity, and specificity of the *Myriapod^®^ NGS BRCA 1-2* solution were calculated and compared to those of the *TSO500HT* and *SOPHiA DDM™ HRD Solution* kits.

### 2.7. Data Analysis

Sequencing data were processed and interpreted using the *Myriapod^®^ NGS* data analysis software (v 5.0.11), a CE-marked in vitro diagnostic application for targeted NGS assays within the Diatech NGS Applications portfolio. The software automatically generates an initial variant report, incorporating SNVs, *indels*, and CNV analysis. For CNV detection, it plots each gene on an independent chart as a graphical visualization, assigning a copy-number score for each exon or amplicon.

## 3. Results

A total of 40 FFPE samples, previously tested by reference methods for *BRCA* CNVs (Table 1), were screened using the *Myriapod^®^ NGS BRCA1/2* panel in combination with the *Myriapod^®^ NGS* data analysis software (v 5.0.11). The performance of CNV detection was assessed at three distinct levels:

(a) Graphical visualization and interpretation of CNV plots;
(b) CNV calling by the *Myriapod^®^ NGS* data analysis software (v 5.0.11);
(c) Final interpretation and reporting of CNV status, as a decision-making result integrating the two previous analysis levels.

### 3.1. Myriapod^®^ NGS Data Results

#### 3.1.1. BRCA CNV-Negative Samples

Based on the graphical visualization and interpretation of CNV status, out of the 23 CNV-negative samples, 16 could be considered negative for both genes. Four samples were CNV-negative for *BRCA2* but showed a potential CNV in *BRCA1*. One sample was CNV-negative for *BRCA1* with an inconclusive CNV result in *BRCA2*, while another was CNV-negative for *BRCA2* with an inconclusive CNV result in *BRCA1*. Finally, one sample was negative for *BRCA1* and showed an “other CNV” in *BRCA2*.

According to the CNV calling performed by the *Myriapod^®^ NGS* data analysis software (v 5.0.11), seven samples were classified as *CNV Not Positive* for both genes. Nine samples were *CNV Not Positive* for *BRCA2* but showed a *Potential CNV* in *BRCA1*. Three samples were identified as *Potential CNV* for both genes, and four samples showed a *Potential CNV* in *BRCA2* and were *CNV Not Positive* for *BRCA1*.

The final interpretation and reporting of CNV status, based on both graphical visualization and software-based CNV calling, led to the classification of 18 samples as *negative* for *BRCA* CNVs, 3 samples as showing a *Potential CNV* in *BRCA1*, 1 sample as *Inconclusive* in *BRCA2*, and 1 sample with a potential CNV in *BRCA2* (Table 2).

Considering the final interpretation and reporting of CNV results, out of the 23 negative samples, 18 would be considered completely negative, and 5 would be referred for confirmatory testing.

#### 3.1.2. BRCA CNV-Positive Samples

Based on graphical visualization and interpretation of CNV status, among the 17 CNV-positive samples, 10 were confirmed as *Positive*. In two samples, a CNV was confirmed in *BRCA2*, while a *Potential CNV* was suspected in *BRCA1*. In one sample, the CNV was confirmed in *BRCA1* and considered *Inconclusive* in *BRCA2*. In two samples, the CNV was not confirmed in *BRCA1* and was *Inconclusive* in *BRCA2*. In another sample, a CNV was confirmed in *BRCA1*, and an additional CNV was suspected in *BRCA2*. Finally, one sample was considered a complete CNV calling failure in both genes. According to CNV calling by the Diatech software, one sample was classified as *failed*.

In nine samples, the expected CNV was correctly identified. Of these, five also showed a *Potential CNV* in the other gene, where a negative result was expected. In the remaining four, only the expected CNV was detected. In seven samples, the expected CNV was not detected, but a *Potential CNV* was identified in the other gene.

The final interpretation and reporting of CNV status, integrating graphical visualization with software-based calling, resulted in 13 samples being classified as definitively CNV-positive. Two samples were negative for the expected CNV but showed inconclusive findings in other gene. One sample was interpreted as *Inconclusive* for CNVs in both target genes, and one sample was classified as *failed* (Table 2).

Considering the final interpretation and reporting of CNV results, out of the 17 positive samples, 13 would be considered *Positive*, 3 would be considered *Negative*, and 1 would be considered *Inconclusive.*

Overall, under these analytical conditions, the *Myriapod^®^ NGS BRCA1/2* pipeline shows a sensitivity, specificity, and accuracy of about 80% compared to the reference assays (Table 3A).

### 3.2. CNV Calling in a Simulated Diagnostic Scenario (One BRCA CNV-Positive vs. Nine BRCA CNV-Negative Samples in the Same NGS Run)

#### 3.2.1. BRCA CNV-Negative Samples

Based on the graphical visualization and interpretation of CNV status, out of the 23 CNV-negative samples, 22 could be considered negative for both genes. One sample was *Negative* for *BRCA2* but showed a *Potential CNV* in *BRCA1* (Table 4).

According to the CNV calling performed by the Diatech software, 13 samples were classified as *CNV Not Positive* for both genes. Four samples were *CNV Not Positive* for *BRCA1* but showed a *Potential CNV* in *BRCA2*. Four samples showed a *Potential CNV* in *BRCA2* and were *CNV Not Positive* for *BRCA1*. Two samples were identified as *CNV Positive* for both genes (Table 4).

The final interpretation and reporting of CNV status, integrating both graphical visualization and software- based CNV calling, classified 22 samples as *Negative*, and 1 sample as showing a *Potential CNV* in *BRCA1* while being CNV *Negative* in *BRCA2*. Based on this final interpretation and reporting, among the 23 samples initially classified as CNV-negative, 22 were considered definitively negative, and 1 sample was recommended for confirmatory testing (Table 4).

#### 3.2.2. BRCA CNV-Positive Samples

Based on the graphical visualization and interpretation of CNV status, among the 17 CNV-positive samples, 14 were confirmed as *Positive.* One sample showed a *confirmed CNV* in *BRCA1* with an *Inconclusive* result in *BRCA2*, while another sample lacked the expected CNV in *BRCA1* and was also *Inconclusive* in *BRCA2* (Table 4).

According to the CNV calling performed by the Diatech software, one sample was classified as *failed*. In 12 samples, the expected CNV was correctly identified. Of these, four also showed a *Potential CNV* in the other gene, where a negative result was expected. In the remaining eight samples, only the expected CNV was detected. Four samples did not show the expected CNV in *BRCA1* but instead showed a CNV in *BRCA2 (*Table 4*)*.

The final interpretation and reporting of CNV status, integrating graphical visualization with software-based calling, resulted in 15 samples being classified as definitively CNV-positive. One sample was interpreted as *Negative* in *BRCA1* (despite the expected CNV) and *Inconclusive* in *BRCA2*. One sample was classified as failed (Table 4).

Overall, under these analytical conditions, the Diatech pipeline showed a sensitivity, specificity, and accuracy of about 95% when compared to the reference assays (Table 3B).

### 3.3. Sequencing Metrics and Performance

**Figure 1:**
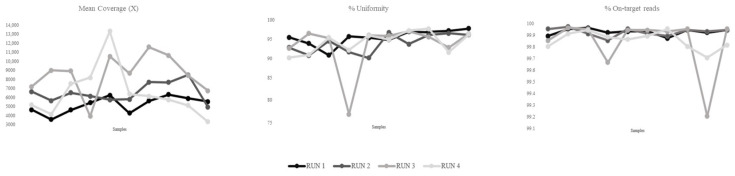
Graphical representation of *mean Coverage (X), on-target reads (%),* and *uniformity (%),* used to assess sequencing performance intra- and inter-runs.

The distribution of sequencing quality metrics across individual samples in different runs is shown in Figure 1. Mean coverage was consistently high. Specifically, each sequenced sample achieved an average coverage exceeding 4000×, indicating sufficient depth in line with the expected performance for tumor sequencing. Coverage uniformity further confirmed efficient and balanced target enrichment across the panel.

The percentage of on-target reads showed minimal variation between samples, demonstrating the high specificity and robustness of the protocol, and confirming that the vast majority of reads were aligned to the intended targets, resulting in reliable and uniform coverage.

Among the three metrics reported in the table, the mean coverage, coverage uniformity, and on-target read percentage, coverage uniformity is the one that most strongly impacts the accuracy and reliability of CNV detection across different samples and sequencing runs.

Mean coverage was maintained at elevated levels across all four runs, with a total average of 6680 ± 1346×. The mean uniformity across all samples was 94.9% ± 0.88, indicating efficient and balanced coverage of the targeted regions. The percentage of on-target reads was remarkably high across all runs, with minimal variability, with a total mean of 99.8% ± 0.05. Taken together, these results demonstrate a high level of technical reliability across runs, with all quality metrics falling within expected and acceptable thresholds. Although this analysis was performed on a relatively small sample set, the data reflect the robustness and high quality of the sequencing process. Collectively, these metrics demonstrate a strong technical reliability across runs, with all quality parameters well within expected and acceptable thresholds.

## 4. Discussion

This study evaluated the analytical performance of different NGS strategies for the detection of *BRCA* CNVs in FFPE samples from OC patients. Specifically, we focused on the concordance of CNV calls between two hybrid capture-based protocols and the amplicon-based *Diatech Myriapod^®^ NGS BRCA1/2* panel. Particular attention was given to the clarity and reliability of result interpretation, as well as the practical feasibility of integrating these methods into routine clinical diagnostics. A key emphasis was placed on the crucial role of bioinformatics pipelines in enabling accurate and robust CNV detection, particularly when handling FFPE-derived DNA, which is often degraded and affected by tumor heterogeneity [14]. Amplicon-based sequencing protocols are widely used in clinical diagnostics due to their high efficiency in detecting SNVs and *indels* [15]. However, several studies have highlighted the limitations of these methods in accurately detecting CNVs, particularly in FFPE samples [12].

An interesting aspect was the strategy used to evaluate the *Diatech Myriapod^®^ NGS BRCA1/2* solution. Specifically, the bioinformatics pipeline was first intentionally stressed by assessing CNV calling under diagnostic conditions, with up to five *BRCA* CNVs in a run of 10 samples. Subsequently, a more clinically realistic scenario was simulated, in which only one CNV might be present in an NGS run of 10 OC samples. In both analytical conditions, the performance of the pipeline was satisfactory. In the second analysis mode, CNV calling showed 96% accuracy in correctly identifying CNVs. Among the positive cases, excluding one sample that failed sequencing (ID: 9), only one case (ID: 17) was missed, while all the remaining CNVs were correctly identified (Table 4). We recognize that this limitation may arise particularly in the context of somatic CNVs, where different NGS strategies can show variable detection performance, especially due to amplicon-specific dynamics within each run. However, missing a somatic CNV is generally considered less critical than failing to identify a germline CNV, which has significant implications for patient management and familial risk assessment [16]. In this contest, we emphasize that all germline rearrangements were successfully identified by the new NGS strategy (Table 4). Regarding the clinical classification, all CNVs were reported as oncogenic according to the OncoKB Database (Table 1). In addition, most of these are currently reported in germline databases, such as ClinVar https://www.ncbi.nlm.nih.gov/clinvar/ (accessed on 3 June 2025) and LOVD “https://databases.lovd.nl/shared/genes” (accessed on 3 June 2025), and classified as pathogenic. The clinical applicability of these variants has allowed our patients to benefit from treatment with PARP inhibitors. Furthermore, the identification of a germline variant enables genetic testing for the patient’s closest relatives.

**Figure 2:**
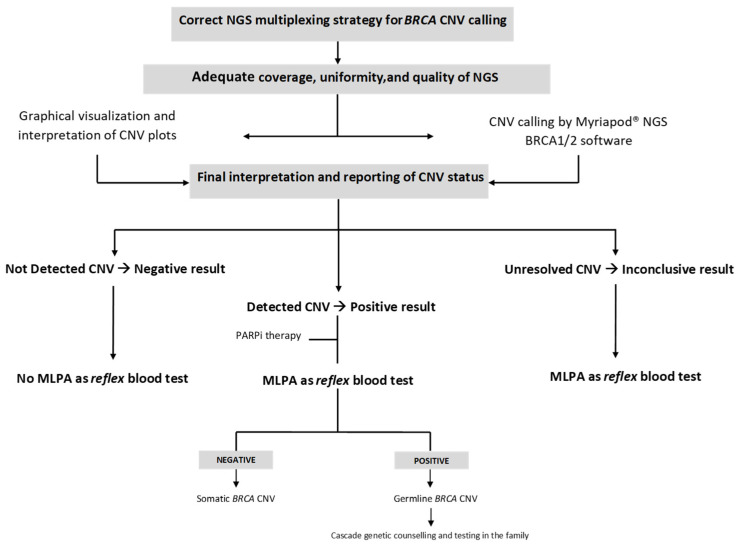
Strategy for interpreting CNV calls from NGS data in FFPE samples, optimized for real-world clinical implementation.

From a diagnostic perspective, our findings suggest that graphical visualization and software-based interpretation should be considered complementary tools. Therefore, a multi-step approach combining algorithmic CNV calling, graphical visualization, and expert review is confirmed as the most reliable strategy, particularly for CNV detection in amplicon-based protocols using FFPE samples (Figure 2). It is essential to note, however, that the manufacturer’s instructions require that samples automatically classified by the software as potentially CNV-positive must always be confirmed by an orthogonal method.

The reliability of CNV detection is also intrinsically linked to the quality of sequencing data. Accurate variant calling requires that key quality metrics, such as the mean coverage, coverage uniformity, and on-target read percentage, meet established thresholds. When these metrics fall below recommended levels, the risk of inconclusive or incorrect calls increases, primarily due to insufficient read depth or uneven read distribution. In our study, sequencing metrics remained consistently high across runs, with mean coverage exceeding 6600× and on-target rates approaching 100%, supporting the technical robustness of the workflow and likely contributing to successful CNV detection.

Among the strengths of this study are the use of real-world clinical FFPE samples, comparison across different NGS platforms, and simulation of practical diagnostic scenarios. However, certain limitations must be acknowledged. First, the sample size was relatively small and may not fully capture the heterogeneity of *BRCA* CNVs observed in routine clinical practice. Second, orthogonal validation methods (e.g., MLPA and qPCR) were not employed for all discordant or borderline cases, which may have introduced uncertainty in result interpretation.

Looking ahead, integrating automated quality control checkpoints and confidence scoring for CNV calls could reduce the burden of manual review while enhancing overall reliability. Furthermore, continued development and validation of dedicated CNV detection algorithms specifically optimized for amplicon-based sequencing will be essential for broader clinical implementation.

## 5. Conclusions

Our study highlights the importance of having validated NGS workflows and bioinformatics pipelines for the accurate detection of *BRCA* CNVs in FFPE tumor samples. The amplicon-based *Myriapod^®^ NGS BRCA1/2* panel in combination with the *Myriapod^®^ NGS data analysis* software (v 5.0.11) proved effective in identifying CNVs, demonstrating strong concordance with hybrid capture-based approaches when combined with optimized bioinformatics analysis, expert interpretation, and reporting.

These elements are essential for the reliable molecular identification of *BRCA* CNVs, which have critical implications for the management of OC patients, including therapeutic decisions involving PARP inhibitors and cascade testing in hereditary cancer syndromes.

For these reasons, it is possible to hypothesize a decision-making workflow, as described in Figure 2, where suspected oncogenic CNVs can guide either a therapeutic approach or germline screening, similar to other PVs in *BRCA* genes. Therefore, the accurate and reliable detection of these alterations remains a fundamental requirement in the genomic evaluation of OC patients.

### Limitations

This study has some limitations. Despite the significant number of *BRCA* CNVs analyzed, all were CNV losses; therefore, we were unable to assess the performance of the evaluated software in detecting *BRCA* CNV gains.

An additional limitation is the lack of detailed information on the CNV calling algorithms used by each platform. As stated in the manuscript, each bioinformatics pipeline is proprietary, which limits transparency regarding the specific parameters or thresholds applied during CNV detection.

## Figures and Tables

**Table 1:** Sample characteristics and molecular results.

ID	Disease	Timing	Age of the Sample	TC (%)	Reference Assay	CNV Status	Detected CNVs *	CNV Status	OncoKB Database Classification ***	Clinical Implication
1	HGSC	PRIMARY	2024	90	TSO500HT	Positive	*BRCA2* exon 2–3 deletion	Somatic	Oncogenic (loss of function)	PARPi treatment
2	HGSC	PRIMARY	2024	90	TSO500HT; SOPHiA DDM HRD	Positive	*BRCA1* exon 19 deletion	Somatic	Oncogenic (loss of function)	PARPi treatment
3	HGSC	PRIMARY	2024	60	TSO500HT; SOPHiA DDM HRD	Positive	*BRCA1* exon 8–11 deletion	Germline **	Oncogenic (loss of function)	PARPi treatment; cascade screening
4	HGSC	PRIMARY	2024	70	TSO500HT; SOPHiA DDM HRD	Positive	*BRCA1* exon 20 deletion	Germline **	Oncogenic (loss of function)	PARPi treatment; Cascade screening
5	HGSC	RELAPSE	2023	90	TSO500HT; SOPHiA DDM HRD	Positive	*BRCA1* exon 2 deletion	Germline **	Oncogenic (loss of function)	PARPi treatment; cascade screening
6	HGSC	RELAPSE	2024	90	SOPHiA DDM HRD	Positive	*BRCA1* exon 16–17 deletion	Germline **	Oncogenic (loss of function)	PARPi treatment; cascade screening
7	HGSC	RELAPSE	2024	70	TSO500HT; SOPHiA DDM HRD	Positive	*BRCA1* exon 4–7 deletion	Germline **	Oncogenic (loss of function)	PARPi treatment; cascade screening
8	HGSC	PRIMARY	2023	90	TSO500HT; SOPHiA DDM HRD	Positive	*BRCA2* exon 9–21 deletion	Somatic	Oncogenic (loss of function)	PARPi treatment
9	ENOC	PRIMARY	2024	90	TSO500HT	Positive	*BRCA1* exon 11 deletion	Somatic	Oncogenic (loss of function)	PARPi treatment
10	HGSC	PRIMARY	2024	95	TSO500HT; SOPHiA DDM HRD	Positive	*BRCA1* exon 2–3 deletion	Somatic	Oncogenic (loss of function)	PARPi treatment
11	HGSC	PRIMARY	2024	60	TSO500HT; SOPHiA DDM HRD	Positive	*BRCA1* exon 19 deletion	Germline *	Oncogenic (loss of function)	PARPi treatment; cascade screening
12	HGSC	RELAPSE	2024	80	SOPHiA DDM HRD	Positive	*BRCA1* exon 2 deletion	Germline **	Oncogenic (loss of function)	PARPi treatment; cascade screening
13	HGSC	RELAPSE	2024	80	SOPHiA DDM HRD	Positive	*BRCA1* exon 2–19 deletion	Somatic	Oncogenic (loss of function)	PARPi treatment
14	HGSC	PRIMARY	2024	80	TSO500HT	Positive	*BRCA1* exon 15 deletion	Somatic	Oncogenic (loss of function)	PARPi treatment
15	HGSC	PRIMARY	2024	55	TSO500HT; SOPHiA DDM HRD	Positive	*BRCA1* whole gene deletion	Germline **	Oncogenic (loss of function)	PARPi treatment; cascade screening
16	HGSC	PRIMARY	2025	30	TSO500HT; SOPHiA DDM HRD	Positive	*BRCA2* exon 11–27 deletion	Somatic	Oncogenic (loss of function)	PARPi treatment
17	HGSC	PRIMARY	2024	80	TSO500HT	Positive	*BRCA1* exon 3–23 deletion	Somatic	Oncogenic (loss of function)	PARPi treatment
18	OCS	RELAPSE	2024	60	TSO500HT; SOPHiA DDM HRD	Negative	-	-	-	-
19	HGSC	PRIMARY	2024	80	TSO500HT; SOPHiA DDM HRD	Negative	-	-	-	-
20	HGSC	PRIMARY	2025	20	TSO500HT	Negative	-		-	-
21	HGSC	PRIMARY	2024	80	TSO500HT; SOPHiA DDM HRD	Negative	-	-	-	-
22	HGSC	PRIMARY	2024	80	TSO500HT	Negative	-	-	-	-
23	CCOC	PRIMARY	2024	80	TSO500HT	Negative	-	-	-	-
24	ENOC	PRIMARY	2024	90	TSO500HT	Negative	-	-	-	-
25	HGSC	PRIMARY	2024	25	TSO500HT	Negative	-	-	-	-
26	HGSC	PRIMARY	2024	80	TSO500HT; SOPHiA DDM HRD	Negative	-	-	-	-
27	HSGC	RELAPSE	2024	95	SOPHiA DDM HRD	Negative	-	-	-	-
28	ENOC	RELAPSE	2024	30	SOPHiA DDM HRD	Negative	-	-	-	-
29	HSGC	RELAPSE	2024	30	SOPHiA DDM HRD	Negative	-	-	-	-
30	HSGC	RELAPSE	2024	35	SOPHiA DDM HRD	Negative	-	-	-	-
31	HGSC	PRIMARY	2023	70	TSO500HT; SOPHiA DDM HRD	Negative	-	-	-	-
32	CCOC	PRIMARY	2023	80	TSO500HT	Negative	-	-	-	-
33	HGSC	PRIMARY	2024	40	TSO500HT; SOPHiA DDM HRD	Negative	-	-	-	-
34	HGSC	PRIMARY	2024	36	SOPHiA DDM HRD	Negative	-	-	-	-
35	HGSC	PRIMARY	2023	80	SOPHiA DDM HRD	Negative	-	-	-	-
36	HGSC	PRIMARY	2025	70	TSO500HT; SOPHiA DDM HRD	Negative	-	-	-	-
37	CCOC	PRIMARY	2025	20	TSO500HT	Negative	-	-	-	-
38	HGSC	PRIMARY	2025	30	TSO500HT; SOPHiA DDM HRD	Negative	-	-	-	-
39	CCOC	PRIMARY	2025	70	TSO500HT	Negative	-	-	-	-
40	HGSC	PRIMARY	2025	25	TSO500HT	Negative	-	-	-	-

* The reference sequences were NG_005905.2/NM_007294.3 and NG_012772.3/NM_000059.3 for BRCA1 and BRCA2, respectively. ** MLPA was performed as a reflex test on peripheral blood samples using the SALSA P002 BRCA1 and SALSA P045 BRCA2 MLPA kits (MRC Holland). *** OncoKB Database https://www.oncokb.org/ (accessed on 3 June 2025). Abbreviations: HGSC: high-grade serous carcinoma; ENOC: endometrioid ovarian cancer, OCS: ovarian carcinosarcoma; CCOC: Clear Cell Ovarian Carcinoma; TC: tumor content.

**Table 2:** *Myriapod^®^ NGS* data results.

ID Samples	Graphical Visualization and Interpretation of CNV Plots		CNV Calling by Myriapod^®^ NGS Data Analysis Software		Final Interpretation and Reporting CNV	
	*BRCA1*	*BRCA2*	*BRCA1*	*BRCA2*	*BRCA1*	*BRCA2*
*BRCA* CNV-negative samples						
18	cCNV	oCNV	Potential CNV	Potential CNV	Negative	oCNV
19	oCNV	cCNV	Potential CNV	CNV Not Positive	Negative	Negative
20	cCNV	cCNV	CNV Not Positive	Potential CNV	Negative	Negative
21	cCNV	cCNV	Potential CNV	Potential CNV	Negative	Negative
22	cCNV	cCNV	Potential CNV	CNV Not Positive	Negative	Negative
23	cCNV	cCNV	Potential CNV	CNV Not Positive	Negative	Negative
24	cCNV	cCNV	Potential CNV	CNV Not Positive	Negative	Negative
25	cCNV	cCNV	CNV Not Positive	CNV Not Positive	Negative	Negative
26	cCNV	cCNV	CNV Not Positive	CNV Not Positive	Negative	Negative
27	oCNV	cCNV	Potential CNV	CNV Not Positive	oCNV	Negative
28	cCNV	cCNV	CNV Not Positive	CNV Not Positive	Negative	Negative
29	cCNV	cCNV	CNV Not Positive	CNV Not Positive	Negative	Negative
30	oCNV	cCNV	Potential CNV	CNV Not Positive	oCNV	Negative
31	cCNV	cCNV	Potential CNV	CNV Not Positive	Negative	Negative
32	cCNV	cCNV	Potential CNV	CNV Not Positive	Negative	Negative
33	cCNV	cCNV	CNV Not Positive	CNV Not Positive	Negative	Negative
34	cCNV	cCNV	CNV Not Positive	CNV Not Positive	Negative	Negative
35	cCNV	cCNV	CNV Not Positive	Potential CNV	Negative	Negative
36	cCNV	cCNV	CNV Not Positive	Potential CNV	Negative	Negative
37	cCNV	cCNV	CNV Not Positive	CNV Not Positive	Negative	Negative
38	iCNV	cCNV	Potential CNV	CNV Not Positive	Negative	Negative
39	cCNV	iCNV	CNV Not Positive	Potential CNV	Negative	iCNV
40	oCNV	cCNV	Potential CNV	Potential CNV	oCNV	Negative
*BRCA* CNV-positive samples						
1	oCNV	cCNV	Potential CNV	Potential CNV	Positive	Positive
2	cCNV	cCNV	Potential CNV	Potential CNV	Positive	Negative
3	cCNV	cCNV	Potential CNV	Potential CNV	Positive	Negative
4	cCNV	cCNV	Potential CNV	CNV Not Positive	Positive	Negative
5	cCNV	cCNV	Potential CNV	CNV Not Positive	Positive	Negative
6	cCNV	cCNV	CNV Not Positive	Potential CNV	Positive	Negative
7	cCNV	oCNV	CNV Not Positive	Potential CNV	Negative	iCNV
8	oCNV	cCNV	Potential CNV	Potential CNV	oCNV	Positive
9	fCNV	fCNV	CNV Failed	CNV Failed	fCNV	fCNV
10	cCNV	cCNV	CNV Not Positive	Potential CNV	Positive	Negative
11	cCNV	cCNV	Potential CNV	CNV Not Positive	Positive	Negative
12	cCNV	iCNV	CNV Not Positive	Potential CNV	Positive	iCNV
13	cCNV	cCNV	CNV Not Positive	Potential CNV	Positive	Negative
14	cCNV	cCNV	Potential CNV	Potential CNV	Positive	Positive
15	ntCNV	iCNV	CNV Not Positive	Potential CNV	Negative	iCNV
16	cCNV	cCNV	CNV Not Positive	Potential CNV	Negative	Positive
17	ntCNV	iCNV	CNV Not Positive	Potential CNV	iCNV	iCNV

Abbreviations: oCNV: other CNV calling in contrast with the reference test; cCNV: confirmed CNV results considering reference tests; fCNV: failed CNV; iCNV: inconclusive CNV; ntCNV: not detected CNV.

**Table 3:** Concordance analysis of CNV calling between the *Myriapod^®^ NGS BRCA1/2* panel and *TSO500HT* and the *SOPHiA DDM™ HRD* assays. (**A**) Primary CNV calling strategy and concordance analysis (5/4 *BRCA* CNV-positive vs. 5/6 *BRCA* CNV-negative samples). (**B**) CNV calling in a simulated *BRCA* diagnostic setting (one *BRCA* CNV-positive vs. nine *BRCA* CNV-negative samples).

(**A**) Primary CNV calling strategy and concordance analysis		
Diatech Myriapod^®^ NGS CNVs final interpretation	TSO500HT/SOPHiA DDM™ HRD	
	CNV-Positive	CNV-Negative
BRCA CNV-Positive	13	5
BRCA CNV-Negative	3	18
Inconclusive	1	
Analytical Performance	Value (%)	
Sensitivity	81.25	
Specificity	78.26	
Positive predictive value	72.22	
Negative predictive value	85.71	
Accuracy	79.49	
(**B**) CNV calling in a simulated BRCA diagnostic setting		
Diatech Myriapod^®^ NGS CNVs final interpretation	TSO500HT/SOPHiA DDM™ HRD	
	CNV-Positive	CNV-Negative
BRCA CNV-Positive	15	1
BRCA CNV-Negative	1	22
Inconclusive	1	
Analytical Performance	Value (%)	
Sensitivity	93.75	
Specificity	95.65	
Positive predictive value	94.87	
Negative predictive value	93.75	
Accuracy	95.65	

**Table 4:** *Myriapod^®^ NGS data analysis* in a simulated *BRCA* diagnostic setting (one *BRCA* CNV-positive vs. nine *BRCA* CNV-negative samples).

ID Samples	Graphical Visualization and Interpretation of CNV Plots		CNV Calling By *Myriapod^®^ NGS* Data Analysis Software		Final Interpretation and Reporting CNV	
	*BRCA1*	*BRCA2*	*BRCA1*	*BRCA2*	*BRCA1*	*BRCA2*
BRCA CNV-negative samples						
18	cCNV	cCNV	CNV Not Positive	Potential CNV	Negative	Negative
19	cCNV	cCNV	CNV Not Positive	Potential CNV	Negative	Negative
20	cCNV	cCNV	Potential CNV	CNV Not Positive	Negative	Negative
21	cCNV	cCNV	CNV Not Positive	Potential CNV	Negative	Negative
22	cCNV	cCNV	CNV Not Positive	CNV Not Positive	Negative	Negative
23	cCNV	cCNV	CNV Not Positive	CNV Not Positive	Negative	Negative
24	cCNV	cCNV	CNV Not Positive	CNV Not Positive	Negative	Negative
25	cCNV	cCNV	CNV Not Positive	CNV Not Positive	Negative	Negative
26	cCNV	cCNV	CNV Not Positive	CNV Not Positive	Negative	Negative
27	cCNV	cCNV	Potential CNV	CNV Not Positive	Negative	Negative
28	cCNV	cCNV	CNV Not Positive	CNV Not Positive	Negative	Negative
29	cCNV	cCNV	CNV Not Positive	CNV Not Positive	Negative	Negative
30	cCNV	cCNV	Potential CNV	CNV Not Positive	Negative	Negative
31	cCNV	cCNV	CNV Not Positive	CNV Not Positive	Negative	Negative
32	cCNV	cCNV	CNV Not Positive	CNV Not Positive	Negative	Negative
33	cCNV	cCNV	CNV Not Positive	CNV Not Positive	Negative	Negative
34	cCNV	cCNV	CNV Not Positive	CNV Not Positive	Negative	Negative
35	cCNV	cCNV	CNV Not Positive	CNV Not Positive	Negative	Negative
36	cCNV	cCNV	CNV Not Positive	Potential CNV	Negative	Negative
37	cCNV	cCNV	CNV Not Positive	CNV Not Positive	Negative	Negative
38	cCNV	cCNV	Potential CNV	CNV Not Positive	Negative	Negative
39	oCNV	cCNV	Potential CNV	Potential CNV	oCNV	Negative
40	cCNV	cCNV	Potential CNV	Potential CNV	Negative	Negative
BRCA CNV-positive samples						
1	cCNV	cCNV	CNV Not Positive	Potential CNV	Negative	Positive
2	cCNV	cCNV	Potential CNV	CNV Not Positive	Positive	Negative
3	cCNV	cCNV	Potential CNV	Potential CNV	Positive	Negative
4	cCNV	cCNV	Potential CNV	CNV Not Positive	Positive	Negative
5	cCNV	cCNV	Potential CNV	CNV Not Positive	Positive	Negative
6	cCNV	cCNV	Potential CNV	CNV Not Positive	Positive	Negative
7	cCNV	iCNV	CNV Not Positive	Potential CNV	Positive	iCNV
8	cCNV	cCNV	Potential CNV	Potential CNV	Negative	Positive
9	fCNV	fCNV	CNV failed	CNV failed	fCNV	fCNV
10	cCNV	cCNV	Potential CNV	CNV Not Positive	Positive	Negative
11	cCNV	cCNV	Potential CNV	CNV Not Positive	Positive	Negative
12	cCNV	cCNV	Potential CNV	Potential CNV	Positive	Negative
13	cCNV	cCNV	CNV Not Positive	Potential CNV	Positive	Negative
14	cCNV	cCNV	Potential CNV	Potential CNV	Positive	Negative
15	cCNV	cCNV	CNV Not Positive	Potential CNV	Positive	Negative
16	cCNV	cCNV	CNV Not Positive	Potential CNV	Negative	Positive
17	ntCNV	iCNV	CNV Not Positive	Potential CNV	Negative	iCNV

Abbreviations: oCNV: other CNV calling in contrast to the reference test; cCNV: confirmed CNV results considering reference tests; fCNV: failed CNV; iCNV: inconclusive CNV; ntCNV: not detected CNV.

## Data Availability

The original contributions of this study are fully detailed within the article. For any further information, please contact the corresponding author.

## Abbreviations

CNVs: copy number variations; LGRs: large genomic rearrangements; BRCA: *BRCA1/2*; NGS: next-generation sequencing; FFPE: formalin-fixed paraffin-embedded; OC: ovarian cancer; PVs: pathogenic variants; PARP-1: poly-(ADP-ribose) polymerase; HRD: homologous recombination repair; SNVs: single nucleotide variants; INDELS: insertions/deletions; HRR: homologous recombination repair; GII: Genomic Integrity Index; MLPA: multiplex ligation-dependent probe amplification; SD: standard deviation; CI: confidence interval.
